# In silico analysis of sodium ion permeation mechanisms in transient receptor potential vanilloid 1

**DOI:** 10.1038/s41598-025-29092-1

**Published:** 2025-12-29

**Authors:** Yuki Nagasato, Keisuke Sanematsu, Yuko Kawabata, Shingo Takai, Saya Nakamura, Toshiro Matsui, Noriatsu Shigemura

**Affiliations:** 1 Section of Oral Neuroscience, Graduate School of Dental Science, Kyushu University, 3-1-1 Maidashi, Higashi-ku, Fukuoka, 812-8582 Japan; 2 Oral Health/Brain Health/Total Health Research Center, Graduate School of Dental Science, Kyushu University, 3-1-1 Maidashi, Higashi-ku, Fukuoka, 812-8582 Japan; 3 Research and Development Center for Five-Sense Devices, Kyushu University, 744 Motooka, Nishi-ku, Fukuoka, 819-0395 Japan; 4 Dent-craniofacial Development and Regeneration Center, Graduate School of Dental Science, Kyushu University, 3-1-1 Maidashi, Higashi-ku, Fukuoka, 812-8582 Japan; 5 Department of Bioscience and Biotechnology, Faculty of Agriculture, Graduate School of Kyushu University, 744 Motooka, Nishi-ku, Fukuoka, 819-0395 Japan

**Keywords:** TRPV1, Ion permeation, Molecular dynamics, Protein function predictions, Membrane proteins

## Abstract

Transient receptor potential vanilloid 1 (TRPV1) is a non-selective cation channel activated by heat, acidity and chemical ligands. While molecular dynamics simulations have shed some light on the cation permeation processes of TRPV1, the mechanisms in the native-state structure under near-physiological conditions remain unestablished. Therefore, the present study conducted molecular dynamics simulations of near-full-length human TRPV1 under a membrane potential of − 100 mV. During permeation events, sodium ions transiently interacted with three binding sites within the channel pore and moved toward the intracellular side. Potential of mean force analyses revealed that sodium ions in the selectivity filter reduced the energy barrier at the hydrophobic gate, facilitating permeation through cooperative interactions. Additionally, mutation of N677, a pre-gate binding site residue, reduced permeation events. Interaction analysis demonstrated that this residue plays an important role in efficient permeation by mediating moderately strong interactions with sodium ions through their coordinated water molecules. These findings highlight the importance of sodium ion accommodation at the selectivity filter and its interaction with N677 for ion permeation through TRPV1. Our data provide new insights into the gating and conduction mechanisms of TRPV1 under near-physiological conditions.

**Supplementary Information:**

The online version contains supplementary material available at 10.1038/s41598-025-29092-1.

## Introduction

Nociceptive stimuli are detected by transient receptor potential (TRP) channels, a large superfamily of cation channels^1^. The TRP channel family in mammals comprises 28 members categorized into six subfamilies: TRPV (vanilloid), TRPA (ankyrin), TRPM (melastatin), TRPC (canonical), TRPP (polycystin) and TRPML (mucolipin)^2,3^. The wide range of TRP-associated channelopathies highlights the critical role of TRP channels in maintaining homeostasis within the human body^4^.

The TRPV1 channel, a member of the TRPV subfamily, is expressed abundantly in sensory nerves where it mediates non-selective cation influx^5^ in response to diverse stimuli such as noxious heat (> 42℃), low pH (pH < 6.0) and various ligands including capsaicin^5–7^. TRPV1 channels play important roles in pain and noxious heat sensation^8,9^. Research on the structural basis of the responses to nociceptive stimuli has advanced since the three-dimensional structure of TRPV1 was first elucidated in 2013^10^.

Structural data indicate that TRPV1 is a homotetramer similar to voltage-gated potassium channels (Kv). Each subunit of TRPV1 consists of a large intracellular region and six transmembrane regions: the S1–S4 helices form a voltage gate-like domain, and the S5–S6 helices form the channel pore. The channel pore contains two main gates: an upper gate with a selectivity filter (SF) formed by the GMGD motif, and a lower hydrophobic gate (Gate) that contains a highly conserved residue (Ile-680) within the TRPV subfamily^10^. The SF is flexible enough for small molecules, such as the lidocaine derivative QX-314^11–13^, to pass through and allosterically modulate the Gate^10,13^. Although cryo-electron microscopy-derived structures of TRPV1 have provided insights into the dynamics of the pore region that serves as the ion conduction pathway, the precise mechanisms by which ions permeate the channel pore to achieve rapid flux remain unclear.

Molecular dynamics (MD) simulation has proven to be a powerful tool for elucidating the molecular mechanisms of ion permeation and selectivity in monovalent ion channels such as sodium and potassium channels^14^. Recent MD simulations of the TRPV subfamily^15–19^ have observed a phenomenon known as ‘knock-off’, whereby cations arranged along the channel pore move concertedly toward the intracellular side, with the innermost cation ultimately released. However, to achieve sufficient sampling of various events, the above studies used a non-physiological membrane potential (> 400 mV) applied through an external electric field. These investigations also relied on minimal TRPV1 structures that were constructed by removing the pore turret and the C- and N- terminals from the full-length TRPV1, which may have affected the electrophysiological behavior of the channel^10,13,20^. Recent advances in cryo-electron microscopy have increased the available number of full-length TRPV1 structures in the Protein Data Bank (PDB), including the open-state structures activated by the single modality agonist, resiniferatoxin (RTX)^13,21^. This progress has provided access to more realistic TRPV1 structures for MD simulations.

The aim of this study was to elucidate the ion permeation mechanisms of TRPV1 under near-physiological conditions. To achieve this aim, MD simulations were conducted using the full-length human TRPV1 (hTRPV1) activated by RTX under a membrane potential of − 100 mV.

Our findings demonstrate that sodium ion accommodation within the binding sites at the SF plays a key role in lowering the energy barrier at the Gate region. In addition, moderate interactions between sodium ions and N677, a pre-gate binding site residue, mediated by coordinated water molecules, contribute to the efficient permeation of sodium ions through TRPV1.

## Results

### Permeation of sodium ions in TRPV1

**Fig. 1:**
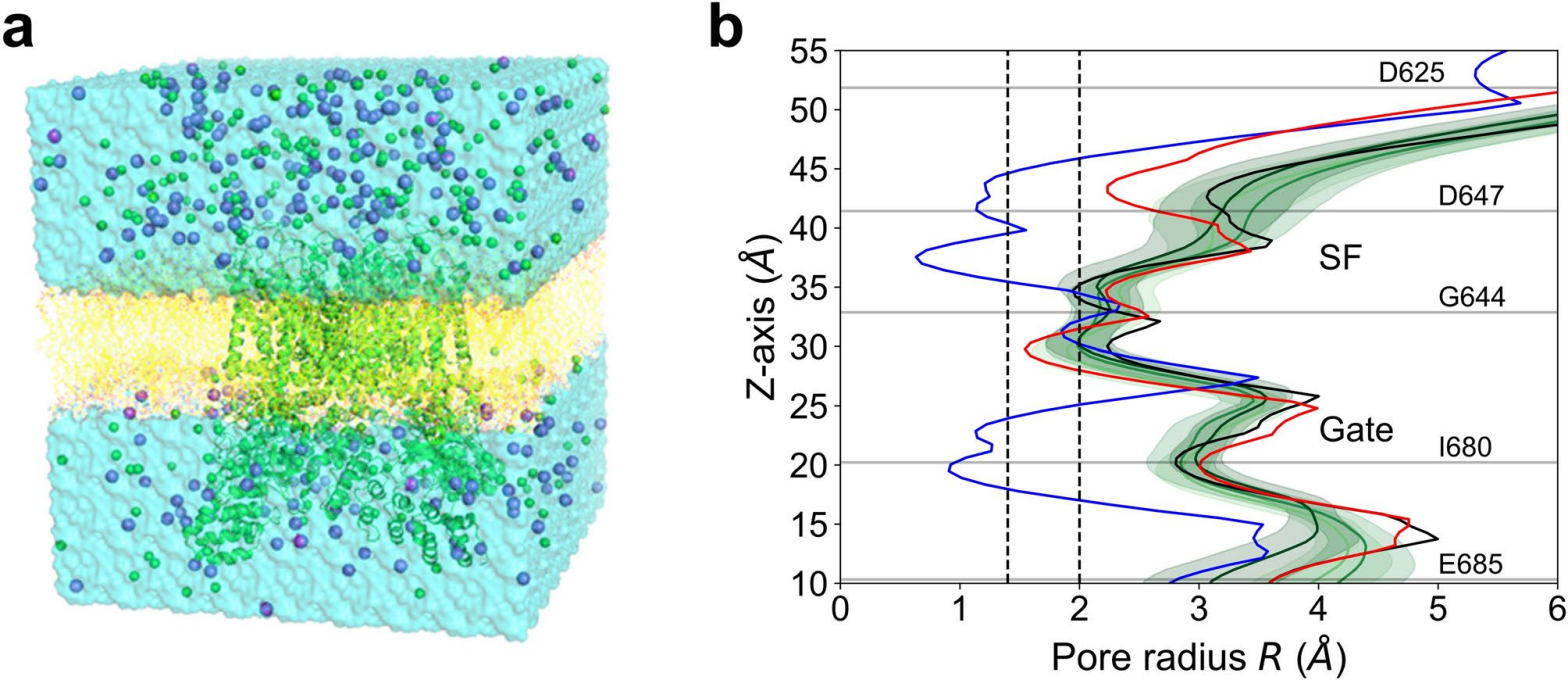
System used for MD simulations and profiles of the TRPV1 channel pore radius. (**a**) A representative simulation system. hTRPV1 was inserted into a POPC bilayer (yellow wire) and solvated in 150 mM NaCl. Sodium ions and chloride ions are shown as purple spheres and green spheres, respectively. Water molecules are shown as the cyan surface. (**b**) The pore radius along the z-axis was calculated using the HOLE program. The gray lines indicate the average positions of D625, G644, D647, I680 and E685 calculated by MD run 1. The average pore radius obtained by the three MD runs are shown by the green solid lines, and the shaded areas indicate the standard deviations. The closed-state sqTRPV1 bound to RTX (7LQZ), open-state rTRPV1 (7RQY) bound to RTX and open-state rTRPV1 bound to RTX and double-knot toxin (3J5Q) are shown by the blue, black and red solid lines, respectively. The dashed lines at 1.4 Å and 2.0 Å indicate the minimum pore sizes required for the passage of water molecules and hydrated cations, respectively.

Since the structure of hTRPV1 has not yet been experimentally resolved, a model of hTRPV1 was generated by homology modeling using two full-length TRPV1 structures bound to RTX as templates: rTRPV1 (open-state, PDB ID: 7RQY^21^) and sqTRPV1 (closed-state, PDB ID:7LQZ^22^). The modeled open-state hTRPV1 was then embedded into a lipid bilayer composed of POPC and solvated with 150 mM NaCl (Fig. 1a). MD simulations were performed under a membrane potential of − 100 mV, closely mimicking physiological conditions.

Each of the three MD simulations of wild-type TRPV1 ran for 1500 ns, resulting in a total sampling time of 4500 ns. Intermittent permeation of sodium ions was observed during these three simulations, with a total of 100 sodium ions passing through the pore (Table 1, Supplementary Movie 1), while chloride ions did not permeate. RTXs remained bound to the vanilloid pockets of hTRPV1 throughout the simulation, and system stability was confirmed by calculation of the RMSD (Fig. S1a). The HOLE program^23^ was used to examine the shape of the channel pore during the MD simulations. The closed-state sqTRPV1 model exhibited two main sites of narrowing corresponding to the SF and the Gate (Fig. 1b). In all simulations of RTX-bound hTRPV1, the pore radius at the Gate (composed of hydrophobic residues) remained above 2.0 Å, which is sufficiently large for a partially hydrated cation to pass through and is consistent with the radius of the open-state rTRPV1 bound to RTX.

**Table 1:** Number of permeation events in each MD simulation.

	Run 1	Run 2	Run 3
Wild-type (1500 ns)	35	31	34
N677A (500 ns)	3	1	0
N677D (500 ns)	8	6	1
N677Q (500 ns)	3	6	2

**Fig. 2:**
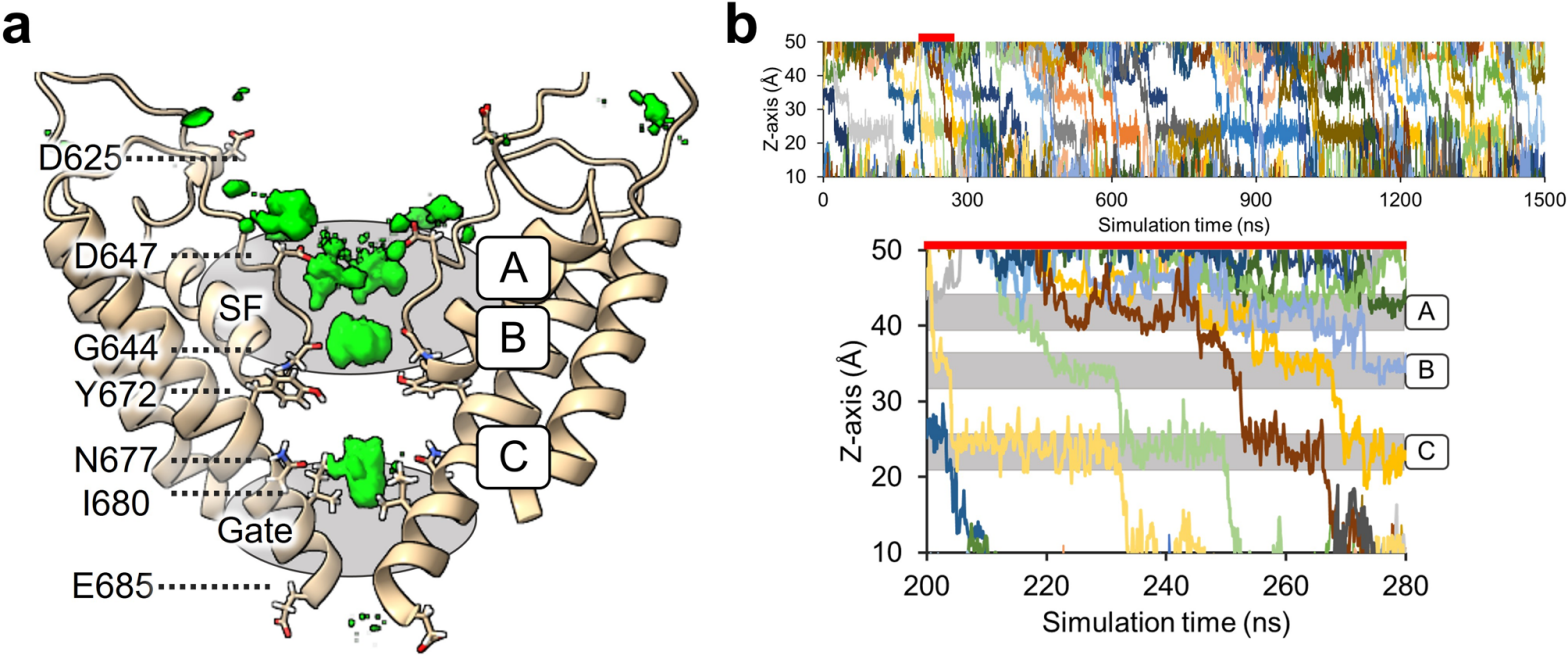
Sodium ion dynamics in the channel pore during permeation events. (**a**) The channel pore region is shown as a tan-colored ribbon diagram. Areas of relatively high sodium ion density (green) were observed near the polar residues located at the site of channel pore narrowing. The threshold for plotting the density was set to 0.005 Å^−3^. Three binding sites (A, B and C) were defined based on the areas of relatively high sodium ion density. The regions corresponding to the SF and Gate are shown as gray ovals. (**b**) The upper panel shows the positions of the permeated sodium ions along the z-axis of wild-type TRPV1 in run 1 of the MD simulation. The section from 200 to 280 ns (red bar) is enlarged in the lower panel.

To gain insight into the movement of sodium ions within the channel pore, we analyzed the sodium ion density within the pore and sodium ion positioning along the z-axis. Evaluation of sodium ion density revealed three binding sites within the channel pore (Fig. 2a). Binding site A consisted of oxygen atoms from both the side chain and backbone of the acidic residue, D647. Binding site B was formed by oxygen atoms from the carboxyl group of G644. Binding site C included oxygen atoms from the side chains of Y672 and N677. The positions of these binding sites were consistent with those of TRPV channels^16–19^. In the MD simulations, binding sites B and C were each occupied by a single sodium ion, whereas binding site A was able to accommodate multiple ions.

During ion permeation events, at least two of the sodium ions arranged along the channel pore (due to interactions with the binding sites) moved simultaneously toward the intracellular side, with the ion at binding site C being released. The sodium ions at binding sites B and C, which each host a single ion, moved cooperatively, whereas the ions at binding site A did not consistently participate in these permeation events (Fig. 2b). The channel conductance calculated from the MD simulations was 35.6 ± 2.2 pS, which is approximately half that obtained from patch-clamp experiments (62.8 ± 4.3 pS)^24^. This discrepancy is not unreasonable given the limitations of using the force field^14^.

### Sodium ion permeation mechanisms

To quantitatively determine the location of the rate-limiting energy barrier within the channel, we calculated the PMF along the channel pore for the system containing a single sodium ion. The 40 Å length within the channel pore along the z-axis, approximately from E685 to D625, was set as the reaction coordinate. A 10-ns MD simulation was performed for each window, totaling 810 ns of MD simulation. The umbrella sampling histograms confirmed that there was sufficient overlap between adjacent windows in each case (Fig. S2). Umbrella sampling with a single sodium ion revealed the energy barrier heights at each binding site: 0.71 kcal/mol at binding site A, 6.37 kcal/mol at binding site B, and 7.52 kcal/mol at binding site C. These results indicate that the largest energy barrier, located beyond binding site C (Gate region), serves as the rate-limiting step for ion permeation.

**Fig. 3:**
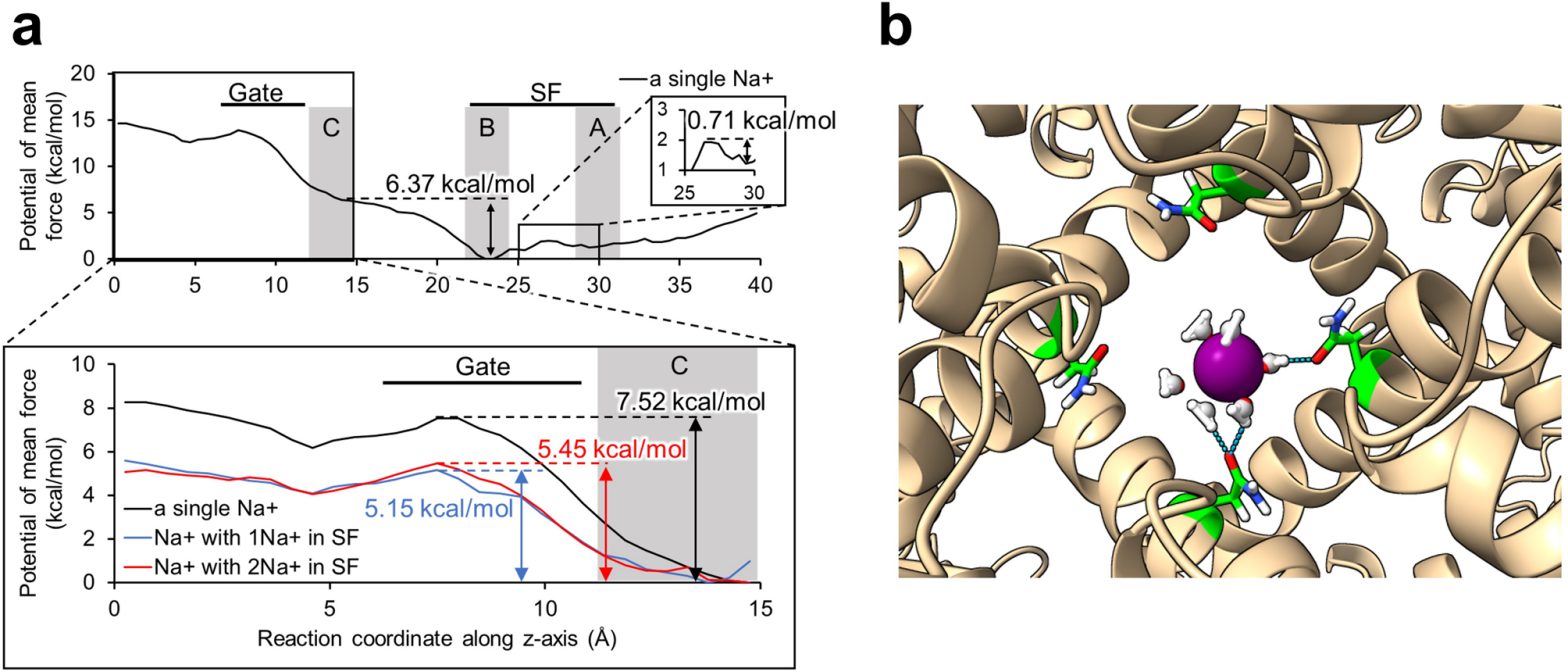
Sodium ion PMF varies depending on the number of sodium ions in the channel pore. (**a**) The PMF of a sodium ion in TRPV1. The upper panel shows the PMF of a single sodium ion within the pore (black line). The boxed regions in the upper panel are magnified in the side and lower panels. In the lower panel, the blue and red lines indicate the PMF of a sodium ion accompanied by one or two sodium ions in the SF, respectively. The positions of binding sites A, B and C are shown by the gray bands. (**b**) The interaction between N677 (in each of the four subunits of TRPV1) and a sodium ion at binding site C. Each N677 is shown as a stick model. The sodium ion is represented by a purple sphere, and water molecules coordinating with the sodium ion are shown as a ball and stick model (oxygen atom: red, hydrogen atom: white). The dashed lines indicate hydrogen bonds.

In addition, to examine how the presence of sodium ions in the SF influences the energy barrier for ion permeation, we calculated the PMF along the channel pore beyond binding site C under conditions containing one or two additional sodium ions in the SF. The results showed that the height of the energy barrier was lower when one sodium ion (5.15 kcal/mol) or two sodium ions (5.45 kcal/mol) were present in the SF region (Fig. 3a). This reduction of the energy barrier induced by additional sodium ions in the SF suggests that interactions between sodium ions at the SF binding sites and binding site C is essential for permeation events. Thus, the accommodation of sodium ions in the SF leads to the efficient and rapid flux through the TRPV1 channel pore.

### Importance of N677 in sodium ion permeation

The energy barrier height is derived from the differences in system stability at each position of the sodium ion, which arises from variations in the interactions between the sodium ion and the hydrophobic residues in the Gate region as well as the residues at binding site C. Interaction analysis revealed hydrogen bonds between water molecules coordinated to the sodium ion and the sidechains of N677 at binding site C (Figs. 3b, S3a).

**Fig. 4:**
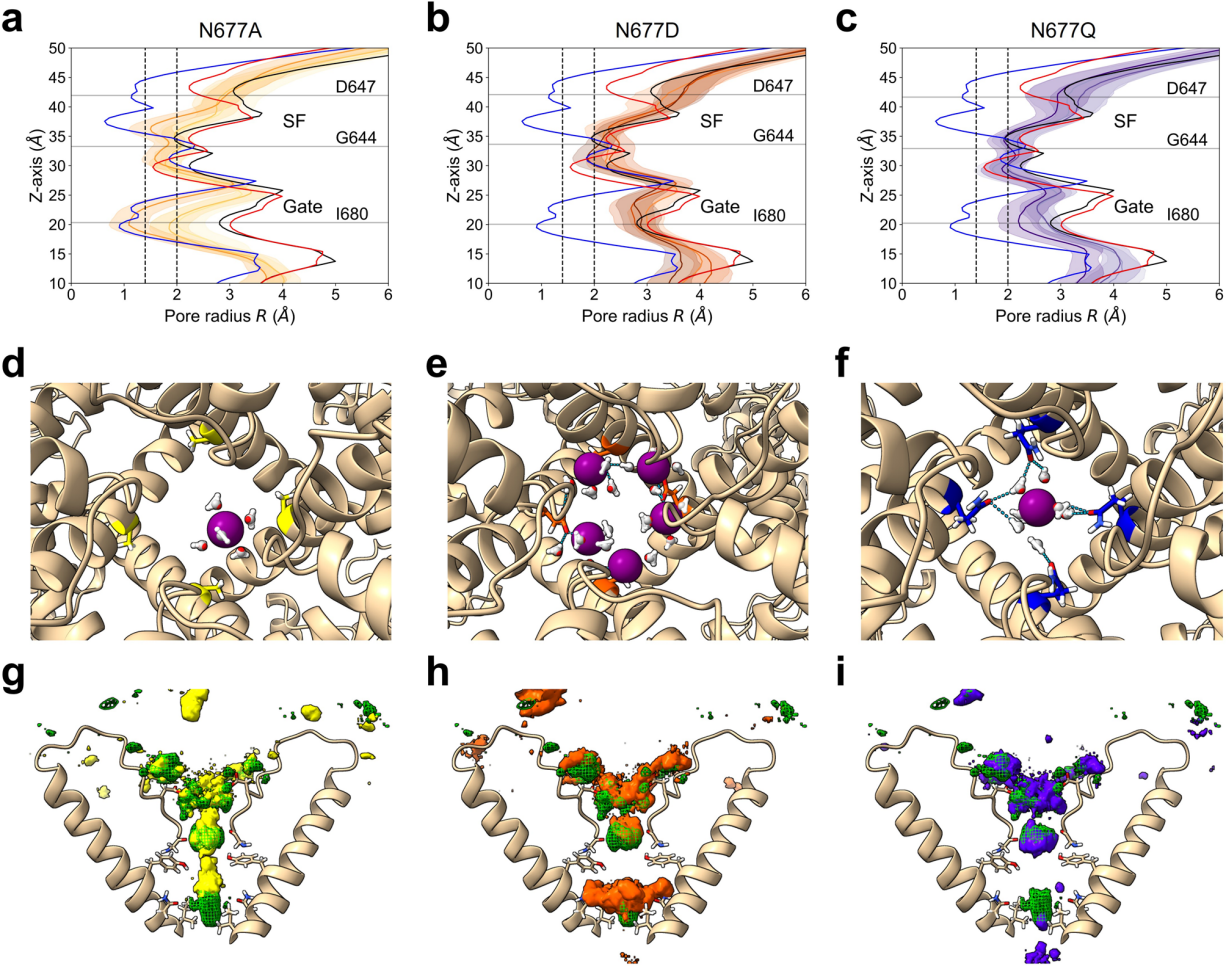
Effects of mutations of N677 on the pore radius and sodium ion dynamics during permeation events. (**a**–**c**) The pore profiles from MD simulations of the TRPV1 mutants N677A, N677D and N677Q are shown in yellow, orange and purple, respectively. The closed-state sqTRPV1 bound to RTX (7LQZ), open-state rTRPV1 bound to RTX (7RQY) and open-state rTRPV1 bound to RTX and double-knot toxin (3J5Q) are shown by the blue, black and red solid lines, respectively. The dashed lines at 1.4 Å and 2.0 Å indicate the minimum pore sizes required for the passage of water molecules and hydrated cations, respectively. (**d**–**f**) Interactions between the sodium ion and binding site C of the N677A, N677D and N677Q mutants of TRPV1. Residue N677 is shown as a stick model, and sodium ions are represented by purple spheres. The water molecules within 3 Å of the sodium ion are shown as a ball and stick model (oxygen atom: red, hydrogen atom: white). Hydrogen bonds are represented by dashed lines. (**g**–**i**) Sodium ion densities in the N677A, N677D and N677Q mutants are shown in yellow, orange and purple, respectively, while that in wild-type TRPV1 is shown by a green mesh. The threshold for plotting these ion densities was set to 0.005 Å^−3^.

The interaction between a sodium ion and polar residue N677 at binding site C is one of the factors that form the largest energy barrier, suggesting that N677 plays a rate-limiting role in the flux of sodium ions. To investigate this hypothesis, mutations were introduced at N677, and MD simulations were performed with the mutated systems for 500 ns. Alanine, aspartic acid and glutamine were selected as the substitutions to represent non-polar, negatively charged and bulky residues, respectively. The stability of each system was confirmed by RMSD calculation (Fig. S1b–d). During the simulations, the pore radius at the Gate region remained larger than 2 Å (open-state) for all mutants, except in run 3 for the N677A system (Fig. 4a–c). The average number of permeation events during each 500-ns MD simulation was lower for the three mutants than for wild-type TRPV1 (Table 1, Fig. S4).

Next, we performed analyses of sodium ion density within the channel pore and sodium ion interactions with binding site C to explore the possible mechanisms underlying impaired permeation in the mutant channels. In the N677A mutant with altered polarity, no interaction was observed between sodium ions and the mutated residue due to its hydrophobicity (Figs. 4d, S3b). This reduction in ion stability led to a shift in the position of peak sodium ion density from N677 in wild-type TRPV1 toward Y672 in the N677A mutant (Fig. 4g). In the N677D mutant with altered charge, a direct and strong interaction was observed between sodium ions and the mutated residue (Figs. 4e, h, S3c), resulting in an increased number of sodium ions at binding site C. These results indicate that the wild-type N677 residue, which is weakly hydrophilic, facilitates the migration of ions to the hydrophobic Gate region by providing an indirect, moderately strong interaction suitable for efficient permeation. In the N677Q mutant, the interactions between Q677 at binding site C and the water molecules coordinated to the sodium ion were comparable to those in the wild-type channel, despite the lower conductance of the mutant channel compared to wild-type TRPV1 (Figs. 4f, i, S3d). The residue densities for wild-type TRPV1 and the N677Q mutant were compared to assess the effect of the bulkiness of the mutated residue. The sidechain of Q677 was found to project into the center of the channel pore, suggesting obstruction of sodium ion permeation (Fig. S3e).

## Discussion

This MD simulation study evaluated sodium ion permeation through a model of an open-state, near-full-length hTRPV1 under near-physiological conditions (150 mM NaCl and a membrane potential of − 100 mV). Intermittent permeation of sodium ions was observed during long MD simulations of the wild-type structure (1500 ns, n = 3; 4500 ns in total), and three binding sites for sodium ions were identified within the channel pore. During permeation events, two or three sodium ions aligned at the binding sites moved cooperatively, and the cooperativity was particularly strong between the two sodium ions bound at binding sites B and C. This well-ordered permeation characterized by binding sites B and C was different from the less-ordered sodium ion permeation observed in MD simulations of TRPV channels under a membrane potential of − 410 mV^16^. A previous study showed that use of a membrane potential less negative than − 410 mV (− 205 mV) achieved a more realistic sodium ion selectivity^16^. Given the above, the permeation dynamics of sodium ions in the present MD simulation, which was carried out under a more physiological membrane potential, may better reflect the actual permeation events in vivo.

The PMF along the channel pore was calculated using umbrella sampling to further understand sodium ion permeation mechanisms in TRPV1. The results showed that the largest energy barrier occurred in the Gate region, and this energy barrier decreased as the number of sodium ions in the SF increased. Previous MD simulations of TRPV1 in the conductive state also concluded that the largest energy barrier was at the Gate region, and the height of this energy barrier was estimated to be 5.2 kcal/mol^25^. This reported value of 5.2 kcal/mol is broadly consistent with the energy barrier estimated in the present study for TRPV1 with two sodium ions (5.15 kcal/mol). These results indicate that at least two sodium ions are required in the channel pore to achieve a normal conductive state in the open-state TRPV1, and this supports the MD simulation finding that cooperative permeation occurs when at least two sodium ions are present in wild-type TRPV1. This cooperative movement of cations during permeation has been observed in other cation channels and has been termed a ‘knock-on’ or ‘knock-off’ mechanism. It has been reported that direct interactions between closely aligned potassium ions in the channel pore enable rapid permeation in potassium channels^26^. On the other hand, the NavAb voltage-gated sodium channel exhibited loose alignment of sodium ions in the channel pore but achieved smooth permeation of sodium ions with the assistance of water molecules^27–29^. In our study, the distance between binding sites B and C in TRPV1 was approximately 10 Å, which is too large for direct interactions between sodium ions. This suggests that the cooperative movement of sodium ions in TRPV1 requires the assistance of water molecules, similar to the mechanism in the NavAb sodium channel. Moreover, our interaction analysis revealed that N677 at binding site C exhibited moderately strong interactions with sodium ions through coordinated water molecules. No electrostatic interactions were observed between sodium ions and hydrophobic residues at the Gate region (Fig. S3a). The difference in the stability of sodium ions at these two positions is thought to be responsible for forming the largest energy barrier.

We investigated the contributions of indirect interactions between N677 and sodium ions by introducing three mutations, N677A, N677D and N677Q, which differ from N677 in terms of hydrophobicity, polarity and bulkiness. MD simulations revealed less frequent permeation events in all three of these mutants than in wild-type TRPV1. N677A caused narrowing of the Gate region, with one simulation exhibiting a fully closed state (gate radius < 1.4 Å) that would prevent the passage of water molecules. A previous in vitro study has reported that the N677A mutant is not a functional channel^30^, supporting the validity of the conformational changes observed in our simulations. The N677D mutation resulted in direct ionic interactions with sodium ions and a high density of sodium ions at binding site C. These findings suggest that a balance between the binding affinity for sodium ions at binding site C and repulsion between sodium ions located at binding sites B and C is critical for permeation events. In wild-type TRPV1, the asparagine residue at position 677 in binding site C provides the appropriate binding affinity for permeation events by interacting with sodium ions through their coordinated water molecules. Indirect interactions with sodium ions through coordinated water molecules were also observed in the N677Q mutant, even though the number of permeation events was lower than that of wild-type TRPV1. Density analysis showed that the sidechains of N677Q projected toward the center of the pore, suggesting that the positively charged amino group of N677Q in each subunit prevented sodium ions from permeating to binding site C. The results of the MD simulations with mutant TRPV1 channels indicate that moderate charge and bulkiness at residue 677 are important for creating binding site C and achieving rapid sodium ion permeation.

N677 is located on the S6 helix that includes an NMLIA sequence that is highly-conserved in TRP channels. Additionally, N677 is conserved in some voltage-gated calcium and sodium channels^31^. The results of our MD simulations highlight the critical role of N677 in regulating the sodium ion movement to enable rapid cation permeation.

## Conclusion

This study conducted MD simulations of near-full-length hTRPV1 under a membrane potential of − 100 mV to elucidate the mechanisms of sodium ion permeation. Observations from conventional MD simulations and PMF calculations using umbrella sampling revealed that the presence of at least two sodium ions aligned along the channel pore leads to rapid sodium ion flux by reducing the energy barrier between binding site C and the Gate region. Furthermore, moderate interactions between N677, a highly conserved residue at binding site C, and sodium ions, mediated by their coordinated water molecules, play a critical role in regulating the frequency of sodium ion permeation.

## Methods

### Molecular modeling

The open-state structure of hTRPV1 was generated by Modeller 10.4^32^ using the rat TRPV1 (rTRPV1) and squirrel TRPV1 (sqTRPV1) structures (PDB ID 7RQY^21^, 7LQZ^22^) as templates and the hTRPV1 sequence (Q8NER1). Specifically, the structure of 7RQY was primarily used as the modeling template, and the missing N- and C-terminal residues were modeled based on 7LQZ. All mutations (N677A, N677D and N677Q) of the asparagine residue at position 677 were introduced using the Rotamers tool in UCSF Chimera^33^. The structure of RTX was obtained from PubChem and optimized by the ab initio quantum chemistry method at the B3LYP/6-31 G(d) level using Gaussian 16 A.03 software^34^. To generate the RTX-hTRPV1 complex, homology models of hTRPV1 and RTX-combined rTRPV1 (7RQY) were superposed based on pairwise alignments using the MatchMaker function in UCSF Chimera. The complex was inserted into a pre-equilibrated palmitoyl-oleoyl-phosphatidylcholine (POPC) bilayer using the Membrane Builder function on the CHARMM-GUI web server (www.charmm-gui.org)^35–37^ and PPM3.0 server^38,39^. The restrained electrostatic potential charges of the ligands were generated by the Antechamber tool in AMBER 20^40^ (Fig. S5 and Table S1). AMBER ff19SB^41^, lipid21^42^ and general AMBER force field 2^43^ were used for proteins, lipids and ligands, respectively. Each system was solvated in an optimal point charge water model^44^. For conventional MD simulations, the solvated system was neutralized with Na^+^/Cl^−^ (150 mM)^45^. For umbrella sampling, one to three sodium ions were placed at specific positions in the channel pore (as detailed in the umbrella sampling section), and chloride ions were added to neutralize the system. The system size reached ~ 160 Å × 160 Å × 170 Å. Energy minimization was performed using the steepest descent method for 2500 steps followed by the conjugated gradient method for 2500 steps.

### MD simulations

Simulations were performed using AMBER 20^40^ for both the equilibration and production processes together with the SHAKE algorithm^46^ based on the protocols provided by the CHARMM-GUI web server. All simulations were conducted under periodic boundary conditions. The short-range cutoff was 9 Å for nonbonded interactions. Long-range electrostatic interactions were calculated by the Particle Mesh Ewald (PME) summation method. The systems were heated to 310.0 K. During the equilibration run, an NVT (constant particle number, volume and temperature) simulation was performed with a 1-fs time step for 250 ps. Subsequently, an NPT (constant particle number, pressure and temperature) simulation was carried out with a 1-fs time step for 0.125 ns and a 2-fs time step for 1.5 ns. Various positional and dihedral restraint potentials were applied with gradual reduction. Thereafter, an additional NPT simulation was conducted with a 2-fs time step for 0.5 ns under an external electric field and without any restraint potentials. Production runs were performed with a 2-fs time step for 1500 ns (wild-type) and 500 ns (mutants), also under an external electric field and without any restraint potentials. To simulate a membrane potential of − 100 mV, an external electric field was applied along the z-axis by setting the AMBER parameter efz to − 0.0135 kcal/mol/Å/e. Each calculation was carried out using the ITO and Genkai supercomputers in the Research Institute for Information Technology, Kyushu University. Three independent simulations were conducted for each experiment.

### Umbrella sampling

Umbrella sampling was used to calculate the potential of mean force (PMF) for three different scenarios, namely one, two or three sodium ions in the channel. The reaction coordinate of the sodium ion was defined as the distance (up to 40 Å for the “a single Na^+^” system, and up to 15 Å in the other two systems) between the ion and the geometric center of the Cα atoms of E685 from all four subunits, measured along the direction parallel to the channel pore (z-axis). The sampled reaction coordinate extended approximately into the extracellular bulk water region but not into the intracellular bulk region. For the PMF calculation with a single sodium ion, the ion was positioned at this geometric center, which served as the starting point of the reaction coordinate. In the case of PMF calculation with two sodium ions, an additional ion was located at binding site B; in the case of PMF calculation with three sodium ions, two additional ions were placed at binding sites A and B. Each system, containing only one to three sodium ions, was neutralized by the addition of chloride ions.

Initially, sodium ions in the system were restrained with a force constraint of 10 kcal/mol/Å^2^. The systems were equilibrated as described above, except for the final 0.5 ns NPT step with an external electric field. During the subsequent ion-pulling and umbrella sampling processes, the positional restraints of 2.5 kcal/mol/Å^2^ were applied to the Cα atoms. When additional sodium ions were present in the SF region, the same positional restraint (2.5 kcal/mol/Å^2^) was also applied to the additional sodium ions. In the ion-pulling processes, the selected sodium ion was pulled at 1 Å/ns and with a force constant of 1.1 kcal/mol/Å^2^, and a series of frames were extracted from the pulling trajectory with a window spacing of 0.5 Å for the ion along the z-direction. The total number of windows reached 81 in the “a single Na^+^” system and 31 in the other two systems. During PMF calculation, only one selected sodium ion was harmonically restrained with a force constraint of 5 kcal/mol/Å^2^. A 10-ns MD simulation was performed for each window, and the last 7 ns of the trajectory were used for the analysis, which was conducted using the weighted histogram analysis method^47^.

### Data analysis

The cpptraj module and MDAnalysis^48,49^ were used for the analysis. The equilibrium state of each simulated model was determined by computing the root-mean-square deviation (RMSD). For calculations of channel pore radii, a series of frames were saved at a framerate of one per 10 ns, and HOLE analysis^23^ was applied to the trajectory. The density of ions in the channel pore was calculated with the Density module in MDAnalysis, applied to the trajectory in which frames were saved at a framerate of one per 0.1 ns. The number of oxygen atoms within 3 Å of the sodium ions was counted by MDAnalysis from the last 300 ns of each MD run. MD movies were created by PyMOL^50^, and hydrogen bonds were detected by Chimera X^51^. Channel conductance (G_TRPV1_) was calculated according to the following equation:

$$G_{TRPV1} = \frac{{N_{permeation} \times Q_{ion} }}{{t_{trajectory} \times V_{membrane} }}$$

where *N*_*permeation*_ represents the number of ions permeated in the MD simulation, *Q*_*ion*_ represents the charge of the ion, *t*_*trajectory*_ refers to the total simulation time, and *V*_*membrane*_ indicates the membrane potential.

## Supplementary Information

Below is the link to the electronic supplementary material.

Supplementary Material 1

Supplementary Material 2

Supplementary Material 3.

## Data Availability

The data supporting the findings of this study are available from the corresponding authors upon reasonable request.
