# Supplementary material for: In silico analysis of sodium ion permeation mechanisms in transient receptor potential vanilloid 1
Source: Sci Rep. 2025 Dec 29;15:44955. doi: 10.1038/s41598-025-29092-1 (PMC12749864; doi:10.1038/s41598-025-29092-1)
Supplement: Supplementary file 1 — Supplementary Material 1 [file 41598_2025_29092_MOESM1_ESM.pdf]

Supplementary Information for:

In Silico Analysis Of Sodium Ion Permeation Mechanisms In Transient Receptor Potential Vanilloid 1

Yuki Nagasato, Keisuke Sanematsu, Yuko Kawabata, Shingo Takai, Saya Nakamura, Toshiro Matsui, Noriatsu Shigemura

The Supplementary Information contains

Supplementary Figures 1–5

Supplementary Table 1

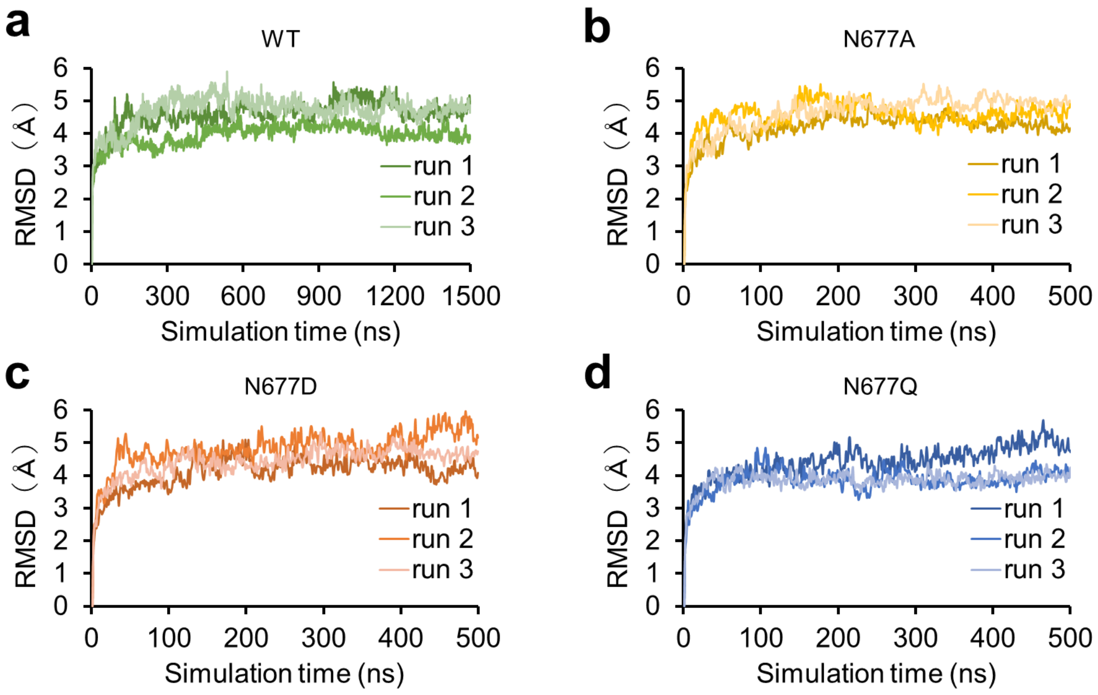

Figure S1: RMSD of the Cα structure of TRPV1 during MD simulations. RMSD of the Cα structure of wild-type (WT) TRPV1 (**a**) and three TRPV1 mutants, N677A (**b**), N677D (**c**) and N677Q (**d**). Three simulations were run in each case.

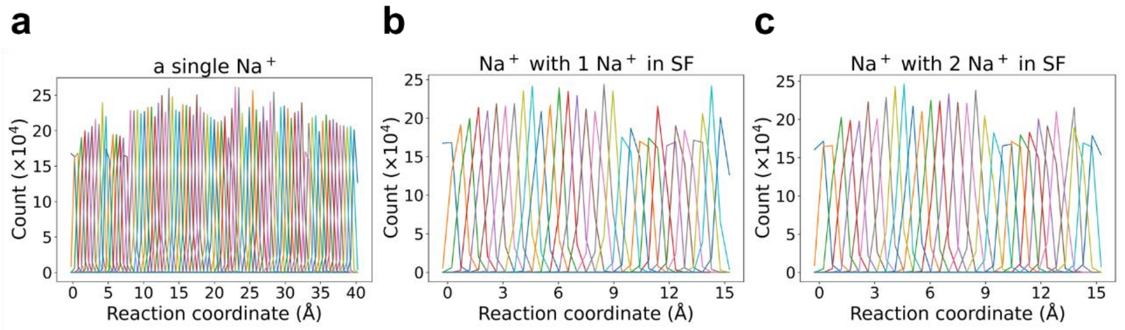

Figure S2: Histograms of umbrella sampling for PMF calculations. Umbrella sampling histograms showing the number (counts) of snapshots, in which the sodium ion existed at the reaction coordinate, for each window. (**a**) A single sodium ion. (**b**) A sodium ion with one other sodium ion in the SF. (**c**) A sodium ion with two other sodium ions in the SF.

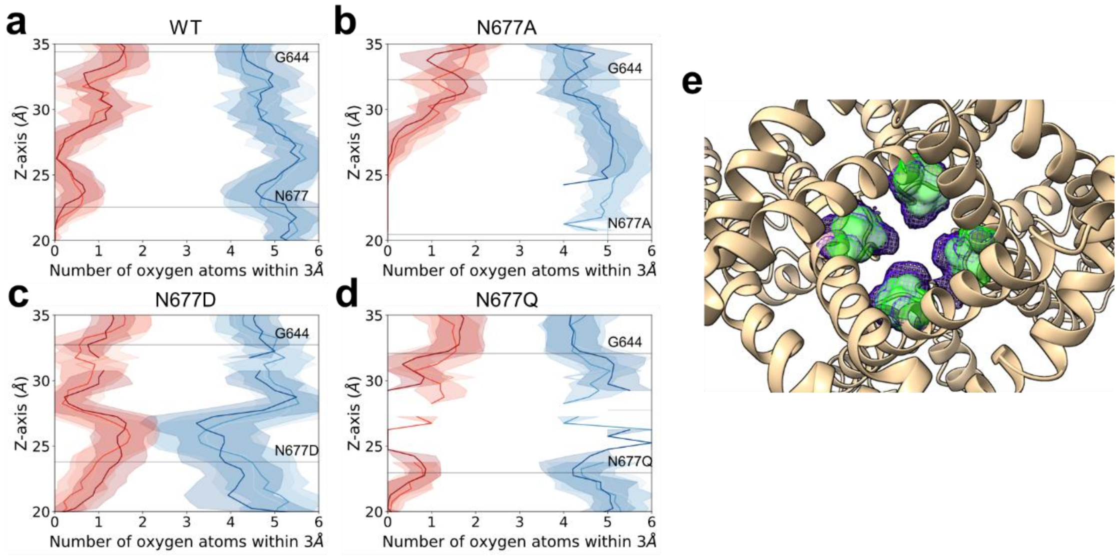

Figure S3: Molecules coordinating with sodium ions in the channel pore and flexibility of the asparagine at position 677. The number of oxygen atoms within 3Å of a sodium ion are shown for wild-type (WT) TRPV1 (**a**) and the TRPV1 mutants, N677A (**b**), N677D (**c**) and N677Q (**d**). Each plot shows the number of oxygen atoms derived from the TRPV1 protein (red line) and from water molecules (blue line). The shaded areas indicate the standard deviations. (**e**) Residue densities for the N677Q mutant (purple mesh) and wild-type TRPV1 (green surface). The threshold for plotting density was set to 0.01 Å^{-3}.

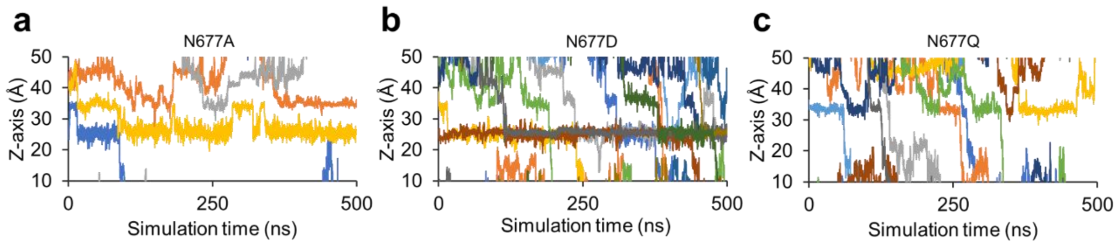

Figure S4: The trajectories of sodium ions transmitted through mutants of hTRPV1. (**a**) N677A mutant. (**b**) N677D mutant. (**c**) N677Q mutant. The transmission trajectories of the sodium ions in one of three simulations are shown for each mutant TRPV1.

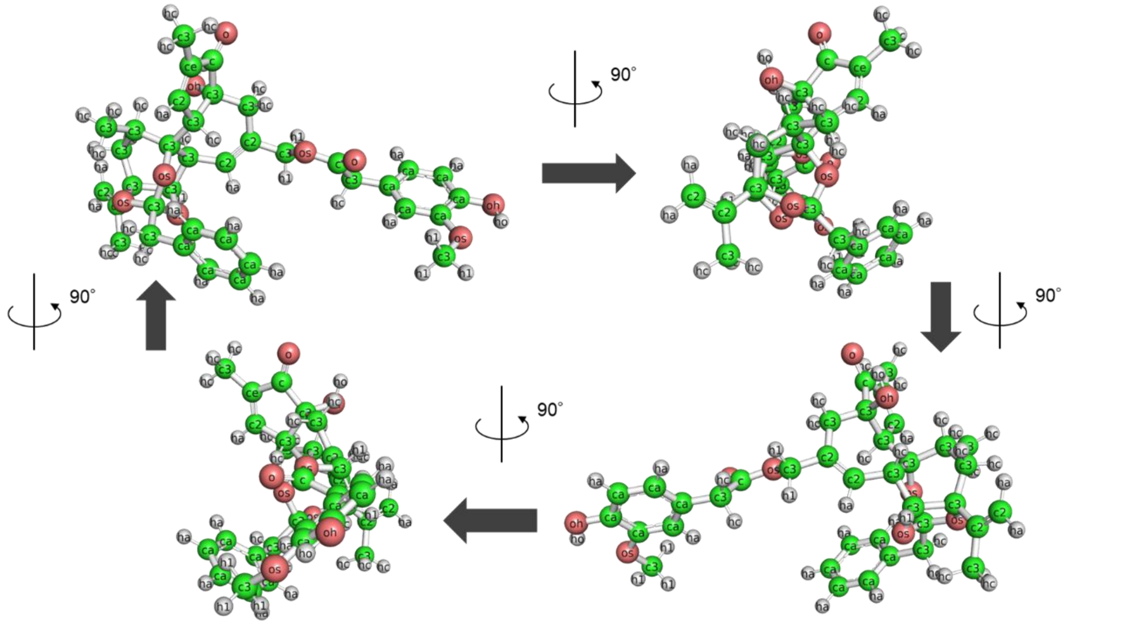

Figure S5: The structure of the ligand resiniferatoxin with a map of the atom types. A ball-and-stick model of resiniferatoxin is shown in multiple orientations, with each atom labeled by its atom type. Oxygen, carbon, and hydrogen atoms are colored red, green, and white, respectively. Atom types correspond to those listed in Table S1.

Table S1: Modified force field parameters for the ligand resiniferatoxin, extracted from the frcmod file generated by antechamber. No modifications were made to the MASS, BOND, ANGLE, and NONBON sections, and therefore, they were not included in the frcmod file.

DIHE

hc-c3-ce-c	1	0.380	180.000	-3.000	same as hc-c3-c2-c2	
hc-c3-ce-c	1	0.000	0.000	-2.000	same as hc-c3-c2-c2	
hc-c3-ce-c	1	1.150	0.000	-1.000	same as hc-c3-c2-c2	penalty score=324.0
hc-c3-ce-c2	1	0.380	180.000	-3.000	same as hc-c3-c2-c2	
hc-c3-ce-c2	1	0.000	0.000	-2.000	same as hc-c3-c2-c2	
hc-c3-ce-c2	1	1.150	0.000	-1.000	same as hc-c3-c2-c2	penalty score=237.0

IMPROPER

c3-ce-c -o	10.5	180.0	2.0	Using general improper torsional angle X- X- c- o	penalty score= 6.0
c -c2-ce-c3	1.1	180.0	2.0	Using the default value	
c3-ce-c2-ha	1.1	180.0	2.0	Same as X -X -ca-ha	penalty score= 47.1 (use general term)
c2-c3-c2-c3	1.1	180.0	2.0	Using the default value	
c2-ha-c2-ha	1.1	180.0	2.0	Same as X -X -ca-ha	penalty score= 47.1 (use general term)
ca-ca-ca-ha	1.1	180.0	2.0	Using general improper torsional angle X- X-ca-ha	penalty score= 6.0
c2-c3-c2-ha	1.1	180.0	2.0	Same as c2-c3-c2-hc	penalty score= 28.3
c3-o -c -os	1.1	180.0	2.0	Same as c3-o -c -oh	penalty score= 8.5
ca-ca-ca-os	1.1	180.0	2.0	Using the default value	
ca-ca-ca-oh	1.1	180.0	2.0	Using the default value	
