# Supplementary material for: In silico analysis of sodium ion permeation mechanisms in transient receptor potential vanilloid 1
Source: Sci Rep. 2025 Dec 29;15:44955. doi: 10.1038/s41598-025-29092-1 (PMC12749864; doi:10.1038/s41598-025-29092-1)
Supplement: Supplementary file 2 — Supplementary Material 2 [file 41598_2025_29092_MOESM2_ESM.docx]

**Supplementary Movie 1**

**Molecular dynamics simulation for the TRPV1 channel.** Overall view and enlarged view of the channel pore region. The boxes in the overall view on the left represent the regions shown in a more detailed view on the right. The structure of TRPV1 is displayed as a green-colored ribbon model, while chloride and sodium ions are represented by green and purple spheres, respectively. In the enlarged view on the right, sodium ions that permeate through TRPV1 are shown as spheres colored differently from purple.
